# Effectiveness of a new cardiac risk scoring model reclassified by QRS fragmentation as a predictor of postoperative cardiac event in patients with severe renal dysfunction

**DOI:** 10.1186/s12872-021-02182-4

**Published:** 2021-07-30

**Authors:** Hyun Jun Cho, Namkyun Kim, Hyeon Jeong Kim, Bo Eun Park, Hong Nyun Kim, Se Yong Jang, Myung Hwan Bae, Jang Hoon Lee, Dong Heon Yang, Hun Sik Park, Yongkeun Cho, Shung Chull Chae

**Affiliations:** 1 Department of Cardiology, Daegu Fatima Hospital, Daegu, Republic of Korea; 2 Department of Medicine, School of Medicine, Kyungpook National University, Daegu, Republic of Korea

**Keywords:** Fragmented QRS, Postoperative cardiac complication, Severe renal dysfunction

## Abstract

**Background:**

It is difficult to evaluate the risk of patients with severe renal dysfunction before surgery due to various limitations despite high postoperative cardiac events. This study aimed to investigate the value of a newly reclassified Revised Cardiac Risk Index (RCRI) that incorporates QRS fragmentation (fQRS) as a predictor of postoperative cardiac events in patients with severe renal dysfunction.

**Methods:**

Among the patients with severe renal dysfunction, 256 consecutive patients who underwent both a nuclear stress test and noncardiac surgery were evaluated. We reclassified RCRI as fragmented RCRI (FRCRI) by integrating fQRS on electrocardiography. We defined postoperative major adverse cardiac event (MACE) as a composite of cardiac death, nonfatal myocardial infarction, and pulmonary edema.

**Results:**

Twenty-eight patients (10.9%) developed postoperative MACE, and this was significantly frequent in patients with myocardial perfusion defect (41.4% vs. 28.0%, *p* = 0.031). fQRS was observed 84 (32.8%) patients, and it was proven to be an independent predictor of postoperative MACE after adjusting for the RCRI (odds ratio 3.279, 95% confidence interval (CI) 1.419–7.580, *p* = 0.005). Moreover, fQRS had an incremental prognostic value for the RCRI (chi-square = 7.8, *p* = 0.005), and to the combination of RCRI and age (chi-square = 9.1, *p* = 0.003). The area under curve for predicting postoperative MACE significantly increased from 0.612 for RCRI to 0.667 for FRCRI (*p* = 0.027) and 23 patients (32.4%) originally classified as RCRI 2 were reclassified as FRCRI 3.

**Conclusions:**

A newly reclassified FRCRI that incorporates fQRS, is a valuable predictor of postoperative MACE in patients with severe renal dysfunction undergoing noncardiac surgery.

## Background

Patients scheduled for surgery and surgeons who are scheduled to perform any surgeries all want to avoid unexpected intraoperative and postoperative complications. Major advanced cardiac events (MACE) such as acute coronary syndrome, pulmonary edema, and cardiac death are principal causes of fatality and mortality in patients with undergoing surgery [1–3]. Therefore, it is hoped that they can be predicted before surgery. To predict the occurrence of perioperative MACE, physicians use score systems such as Revised Cardiac Risk Index (RCRI) to estimate the incidence of perioperative MACE or to investigate the underlying cardiac problems before surgery [4].

Chronic kidney disease in the nonsurgical setting is an established independent predictor of mortality and cardiovascular events [5–7]. Patients with severe renal dysfunction are more vulnerable to postoperative cardiac complications. Patients with impaired renal function were reported to have a much higher incidence of complications than patients with normal kidney function [8]. However it is difficult to perform preoperative cardiac evaluation in patients with severe renal dysfunction due to limitations such as various comorbidities and concerns about deterioration of kidney function due to the use of contrast agents despite these high postoperative events. Myocardial perfusion single-photon emission computed tomography (SPECT) is a useful test to differentiate ischemic heart disease (IHD) in patients with renal dysfunction, but it is also has limitations in that it is not a test that can be easily performed in all facilities.

Previous studies have shown that fragmented QRS (fQRS) on the electrocardiogram (ECG) is associated with myocardial ischemia and/or scar and those patients with fQRS on ECG had a higher incidence of postoperative MACE [9–11]. However the prognostic value of fQRS was not demonstrated in patients with severe renal dysfunction undergoing noncardiac surgery [9–11]. This study aimed to investigate the value of a newly reclassified RCRI that incorporates fQRS (FRCRI) as a predictor of postoperative cardiac event in patients with severe renal dysfunction.

## Materials and methods

Among the patients with severe renal dysfunction, 256 consecutive patients who underwent myocardial perfusion single-photon emission computed tomography for noncardiac surgery at the Kyungpook National University Hospital (Daegu, Korea) between January 2010 and November 2019 were retrospectively enrolled. Among patients with severe renal dysfunction who underwent noncardiac surgery, patients who had not performed myocardial SPECT or had no ECG prior to surgery were excluded from this study. Severe renal dysfunction was defined as dialysis or estimated glomerular filtration ratio calculated by a Modification of Diet in Renal Disease equation below 30 mL/min/body surface area. Clinical characteristics, including age, sex, body mass index, cardiovascular risk factors (hypertension, diabetes, current smoking), and comorbidities (previous IHD, congestive heart failure (CHF), cerebrovascular disease) were identified.

All patients underwent a Tc-99 m MIBI stress/rest gated myocardial perfusion SPECT using a one day protocol. ECG-gated acquisition was performed on stress and rest images at 8 frames per R-R interval, and images were obtained with a dual-head gamma camera (Vertex Plus, ADAC, USA or Prism 3000, Philips, USA). Transaxial tomograms were reconstructed by filtered back-projection in vertical long-axis, horizontal long-axis, and short-axis planes. An independent reader blinded to the ECG findings evaluated the SPECT images. A semiquantitative sum stress score, sum rest score, and sum difference score were calculated on a standard 17-segment, 5-point scale (0 = normal, 1 = equivocal or mildly abnormal, 2 = moderately abnormal, 3 = severely abnormal, and 4 = absent tracer uptake). Myocardial scar was defined by the presence of fixed perfusion defects (> 2 segments). Myocardial ischemia was defined by a total regional sum difference score ≥ 3 without fixed perfusion defects.

**Fig. 1:**
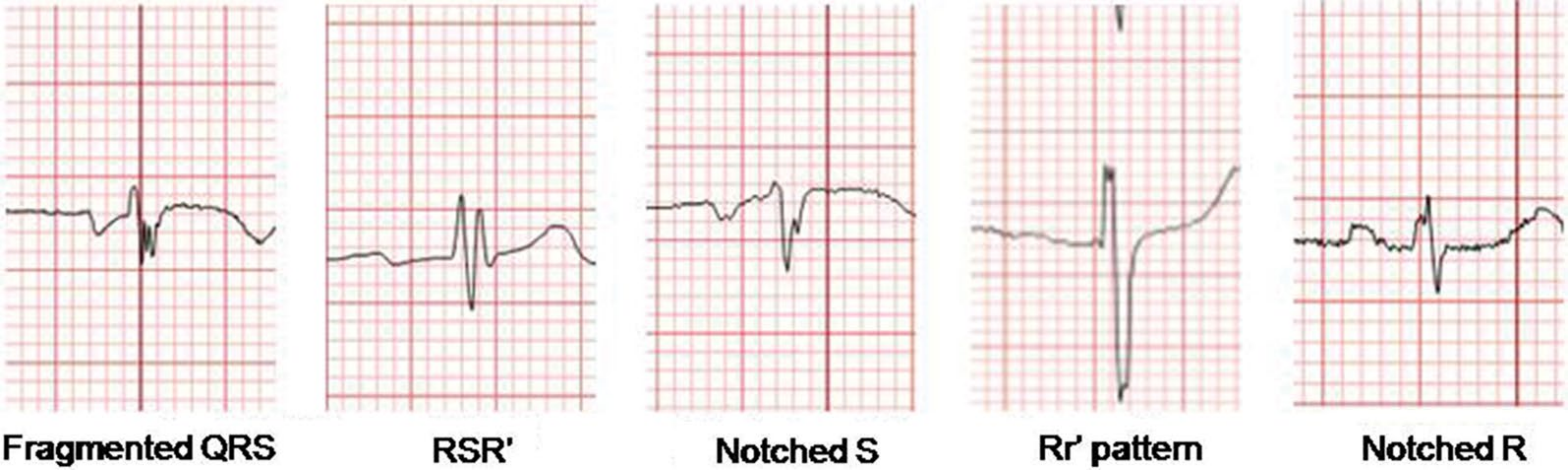
Examples of various fragmented QRS complex morphologies

Among 12-lead ECGs (Filter range, 100 to 150 Hz; 25 mm/s; 10 mm/mV) obtained at rest preoperatively, an ECG obtained on the nearest surgical day was analyzed by two independent cardiologists. An fQRS was defined as changes in QRS morphology (< 120 ms) with diverse RSR′ patterns, i.e., additional R (R′) waves, notching, S waves, or > 1 R′ waves in 2 contiguous leads [10]. In a bundle branch block pattern with a QRS duration of 120 ms, fQRS was defined as different RSR′ patterns, that is, > 2 R waves (R′), > 2 notches in R waves, or S waves in 2 contiguous leads [11]. Those judged to have incomplete bundle branch blocks were excluded. Examples of various fragmented QRS complex morphologies are shown in Fig. 1.

We calculated RCRI scores for each patient by assigning 1 point for the following 6 risk factors: insulin-dependent diabetes; renal insufficiency; and histories of IHD, CHF, cerebrovascular disease, and high-risk surgery [4]. We classified patients as RCRI 1, 2, 3 or ≥ 4 based on the number of risk factors present. Moreover, we then reclassified patients by FRCRI values of 1, 2, 3 or ≥ 4 by summing the RCRI score and the fQRS score. The fQRS was also assigned 1 point. We defined a postoperative MACE as a composite of cardiac death, nonfatal myocardial infarction, and pulmonary edema that occurred during or within 30 days after noncardiac surgery. The study was approved by the Kyungpook National University Institutional Review Board.

Data are expressed as mean ± standard deviation (SD) for continuous variables and as percentages for categorical variables. All comparisons between baseline variables were assessed using the Student’s t-test for continuous variables or with Pearson chi-square test for categorical variables. A multivariate logistic regression model was used to identify independent predictors of postoperative MACE. Incremental factors added to the model at each step were considered significant when differences in log-likelihoods associated with models corresponded to *p* < 0.05. We estimated receiver operating characteristic (ROC) curves and compared areas under curves (with 95% confidence interval (CI)) in corresponding logistic models. The different distributions of RCRI and FRCRI were compared using the area under curve (AUC) of RCRI and FRCRI were using MedCalc. For all analyses, a 2-sided *p* < 0.05 was considered statistically significant. All statistical analyses were performed using the SPSS software version 20.0 for Windows (SPSS Inc, IL, USA) and MedCalc version 19.2.

## Results

Of the 256 consecutive patients (mean age 68.7 ± 12.1 years; 179 men) who underwent myocardial perfusion SPECT for preoperative evaluation of noncardiac surgery, 158 patients (61.7%) were undergoing dialysis. fQRS and myocardial perfusion defect on SPECT were identified in 84 (32.8%) and 95 (37.1%) patients, respectively. Table 1 shows the baseline characteristics according to the presence or absence of fQRS and perfusion defect on SPECT. The fQRS was more common in patients with myocardial perfusion defects and equally, myocardial perfusion defect was common in patients with fQRS on ECG. Previous histories of IHD and CHF, and insulin-dependent diabetes were more common, left ventricular ejection fraction (LVEF) was significantly lower and RCRI score was significantly higher in patients with myocardial perfusion defects. In patients with fQRS on ECG, the average number of fQRS was 2.9 ± 1.2, and there was no difference on the number of leads with fQRS (2.9 ± 1.3 vs. 2.9 ± 1.0, *p* = 0.821) according to the presence or absence of myocardial perfusion defect.

**Table 1:** Clinical and electrocardiographic characteristics with respect to the presence or absence of the fQRS and the myocardial perfusion defect

	All patients (n = 256)	fQRS		*p* value	Perfusion defect on SPECT		*p* value
		Yes (n = 84)	No (n = 172)		Yes (n = 95)	No (n = 161)	
*Demographics*							
Age (year)	68.7 ± 12.1	68.9 ± 10.7	68.6 ± 12.8	0.853	70.5 ± 10.8	67.7 ± 12.8	0.058
Male (%)	179 (69.9)	68 (81.0)	111 (64.5)	0.007	64 (67.4)	115 (71.4)	0.494
Body mass index (kg/m^2^)	22.1 ± 3.1	21.9 ± 2.9	22.2 ± 3.2	0.471	22.1 ± 3.0	22.1 ± 3.1	0.947
*Risk factors and comorbidities*							
Hypertension (%)	213 (83.2)	62 (73.8)	151 (87.8)	0.005	82 (86.3)	131 (81.4)	0.306
Diabetes, all (%)	176 (68.8)	60 (71.4)	116 (67.4)	0.518	71 (74.7)	105 (65.2)	0.112
Diabetes, insulin (%)	78 (30.5)	21 (25.0)	57 (33.1)	0.184	37 (38.9)	41 (25.5)	0.024
Previous IHD (%)	106 (41.4)	41 (48.8)	65 (37.8)	0.093	65 (68.4)	41 (25.5)	< 0.001
Previous CHF (%)	85 (33.2)	26 (31.0)	59 (34.3)	0.593	44 (46.3)	41 (25.5)	0.001
Previous CVD (%)	56 (21.9)	17 (20.2)	39 (22.7)	0.658	21 (22.1)	35 (21.7)	0.945
Current smoking (%)	49 (19.1)	18 (21.4)	31 (18.0)	0.516	17 (17.9)	32 (19.9)	0.657
Dialysis (%)	158 (61.7)	49 (58.3)	109 (63.4)	0.436	50 (52.6)	108 (67.1)	0.022
*Echocardiography*							
LVEF (%)	49.8 ± 12.7	49.7 ± 13.6	49.8 ± 12.3	0.976	45.2 ± 12.4	52.5 ± 12.1	< 0.001
LVEDD (mm)	50.0 ± 7.1	48.7 ± 7.2	50.7 ± 7.0	0.030	51.1 ± 6.5	49.4 ± 7.3	0.076
*Electrocardiography*							
Atrial fibrillation (%)	14 (5.5)	7 (8.3)	7 (4.1)	0.239	3 (3.2)	11 (6.8)	0.212
QRS duration (ms)	97.2 ± 17.7	99.1 ± 17.6	96.2 ± 17.8	0.229	101.8 ± 20.2	94.4 ± 15.5	0.002
ST-T changes (%)	95 (37.1)	33 (39.3)	62 (36.0)	0.614	47 (49.5)	48 (29.8)	0.002
fQRS on the ECG (%)	84 (32.8)	84 (100)	0 (0)	< 0.001	39 (41.1)	45 (28.0)	0.031
Myocardial perfusion defect (%)	95 (37.1)	39 (46.4)	56 (32.6)	0.031	95 (100)	0 (0)	< 0.001
RCRI score	2.4 ± 1.0	2.4 ± 1.0	2.4 ± 1.0	0.980	2.8 ± 1.0	2.1 ± 0.9	< 0.001
FRCRI score	2.7 ± 1.1	3.4 ± 1.0	2.4 ± 1.0	< 0.001	3.2 ± 1.1	2.4 ± 1.0	< 0.001

fQRS = fragmented QRS complex; SPECT = single-photon emission computed tomography; IHD = ischemic heart disease; CHF = congestive heart failure; CVD = cerebrovascular disease; LVEF = left ventricular ejection fraction; LVEDD = left ventricular end-diastolic diameter; RCRI = Revised Cardiac Risk Index; FRCRI = sum of Revised Cardiac Risk Index score and fragmented QRS score

Twenty-eight patients developed MACE (10.9%; cardiac death 3, nonfatal myocardial infarction 18, pulmonary edema not associated with myocardial infarction 7) (Table 2). Patients with postoperative MACE were older and had a higher frequency of previous IHD and took surgery under general anesthesia. The fQRS on ECG was more common in patients with postoperative MACE than in those without (60.7% vs. 29.4%, *p* = 0.001). However, there was no difference on the number of leads with fQRS (2.9 ± 1.0 vs. 2.9 ± 1.2, *p* = 0.845) according to the presence or absence of postoperative MACE in patients with fQRS on ECG. RCRI (2.8 ± 1.0 vs. 2.3 ± 1.0, *p* = 0.047) and FRCRI (3.4 ± 1.1 vs. 2.6 ± 1.1, *p* = 0.001) scores were also significantly higher in patients with postoperative MACE.

**Table 2:** Characteristics of the patients with and without MACE

	MACE (Death/MI/pulmonary edema)		*p* value
	Yes (n = 28)	No (n = 228)	
*Demographics*			
Age (years)	73.7 ± 8.2	68.1 ± 12.4	0.003
Male (%)	19 (67.9)	16 (70.2)	0.801
Body mass index (kg/m^2^)	21.5 ± 3.0	22.1 ± 3.1	0.279
*Risk factors and comorbidities*			
Hypertension (%)	24 (85.7)	189 (82.9)	1.000
Diabetes, all (%)	16 (57.1)	160 (70.2)	0.160
Diabetes, insulin (%)	6 (21.4)	72 (31.6)	0.271
Previous IHD (%)	18 (64.3)	88 (38.6)	0.009
Previous CHF (%)	11 (39.3)	74 (32.5)	0.469
Previous CVD (%)	7 (25.0)	49 (21.5)	0.672
Current smoking (%)	7 (25.0)	42 (18.4)	0.404
Dialysis (%)	15 (53.6)	143 (62.7)	0.347
Echocardiography LVEF (%)	47.3 ± 14.3	50.1 ± 12.5	0.278
LVEDD (mm)	50.0 ± 6.3	50.0 ± 7.2	0.987
*Electrocardiography*			
Atrial fibrillation (%)	2 (1.5)	12 (5.3)	0.656
QRS duration (ms)	94.6 ± 20.4	97.5 ± 17.4	0.429
ST-T changes (%)	14 (50)	81 (35.5)	0.135
fQRS on the ECG (%)	17 (60.7)	67 (29.4)	0.001
Myocardial perfusion defect (%)	14 (50.0)	81 (35.5)	0.135
High surgical risk	7 (25.0)	24 (10.5)	0.057
General anesthesia (%)	19 (67.9)	75 (32.9)	< 0.001
RCRI score	2.8 ± 1.0	2.3 ± 1.0	0.047
FRCRI score	3.4 ± 1.1	2.6 ± 1.1	0.001

MACE = major advanced cardiac events; MI = myocardial infarction; IHD = ischemic heart disease; CHF = congestive heart failure; CVD = cerebrovascular disease; LVEF = left ventricular ejection fraction; LVEDD = left ventricular end-diastolic diameter; fQRS = fragmented QRS complex; RCRI = Revised Cardiac Risk Index; FRCRI = sum of Revised Cardiac Risk Index score and fragmented QRS score

**Fig. 2:**
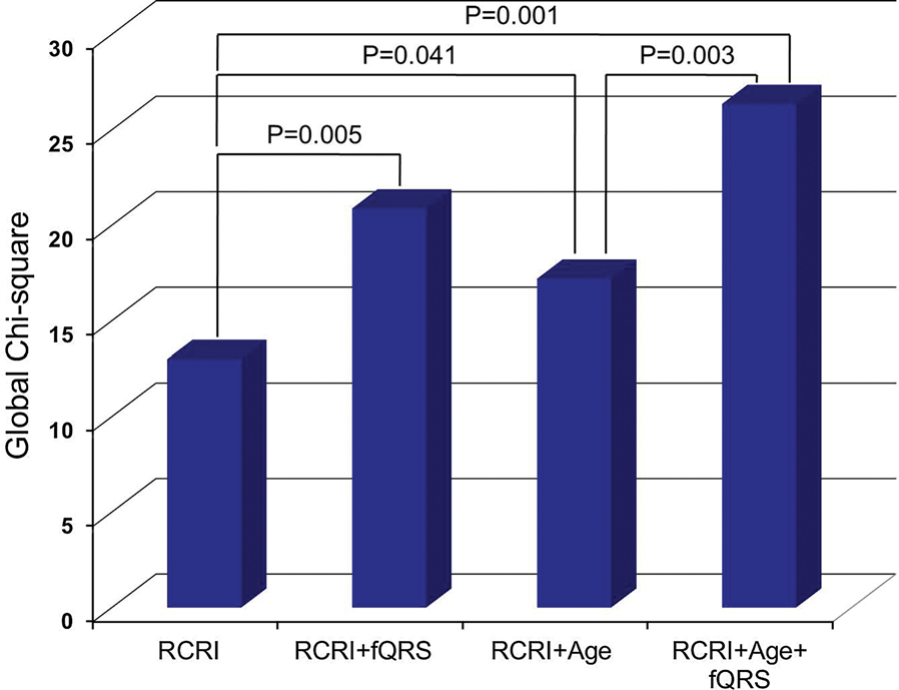
Incremental value of fQRS as determined by a logistic regression model. fQRS added value to RCRI and to the RCRI/Age per year combination. fQRS = fragmented QRS complex, RCRI = Revised Cardiac Risk Index

By multivariate logistic regression analysis, fQRS (odds ratio (OR) 3.279, 95% CI 1.419 to 7.580, *p* = 0.005) was an independent predictor of postoperative MACE after adjusting for components of RCRI (Table 3). The fQRS (OR 3.699, 95% CI 1.563 to 8.754, *p* = 0.003) was still an independent predictor of postoperative MACE after adjusting for the RCRI and age per year. Moreover, fQRS had an incremental prognostic value to RCRI (chi-square = 7.8, *p* = 0.005), and to the combination of RCRI and age (chi-square = 9.1, *p* = 0.003) (Fig. 2).

**Table 3:** Multivariate logistic regression analysis for predicting MACE

	Model 1^a^			Model 2^b^		
	OR	95% CI	*p* value	OR	95% CI	*p* value
Insulin-dependent diabetes	0.623	0.230–1.686	0.352	0.736	0.266–2.039	0.556
Previous IHD	2.617	1.102–6.215	0.029	2.259	0.938–5.439	0.069
Previous CHF	1.388	0.582–3.312	0.460	1.551	0.641–3.750	0.330
Previous CVD	1.285	0.486–3.401	0.613	1.103	0.415–2.934	0.844
High risk surgery	2.820	1.001–7.947	0.050	3.211	1.110–9.292	0.031
fQRS on the ECG	3.279	1.419–7.580	0.005	3.699	1.563–8.754	0.003
Age per year				1.052	1.005–1.100	0.028

MACE = major advanced cardiac events; IHD = ischemic heart disease; CHF = congestive heart failure; CVD = cerebrovascular disease; fQRS = fragmented QRS complex

^a^Model 1 was adjusted for the revised cardiac risk index and the fQRS on ECG

^b^Model 2 was adjusted for the revised cardiac risk index, the fQRS on ECG and age

**Fig. 3:**
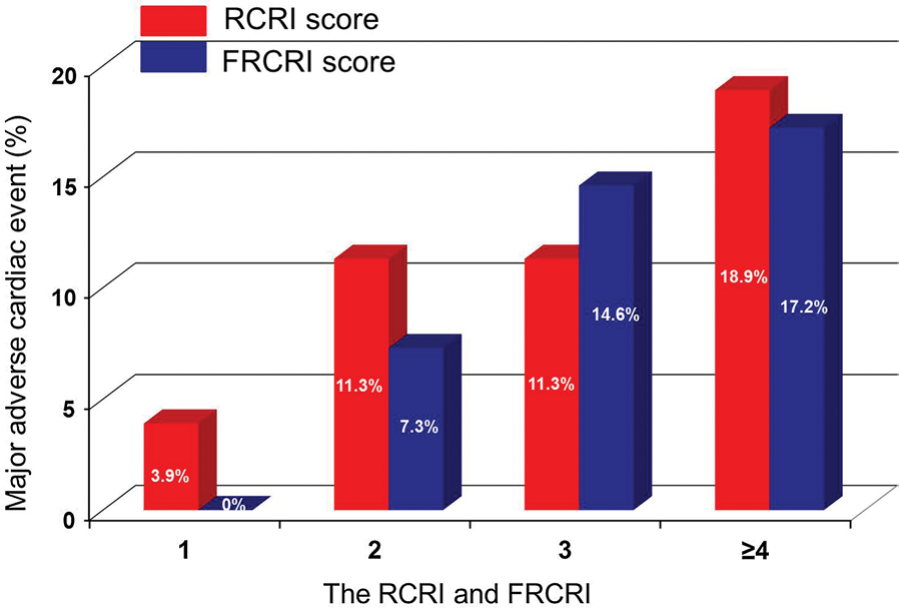
Incidence of MACE according to the RCRI and FRCRI scores. MACE = major adverse cardiac events, RCRI = Revised Cardiac Risk Index, FRCRI = sum of Revised Cardiac Risk Index score and fragmented QRS score

**Fig. 4:**
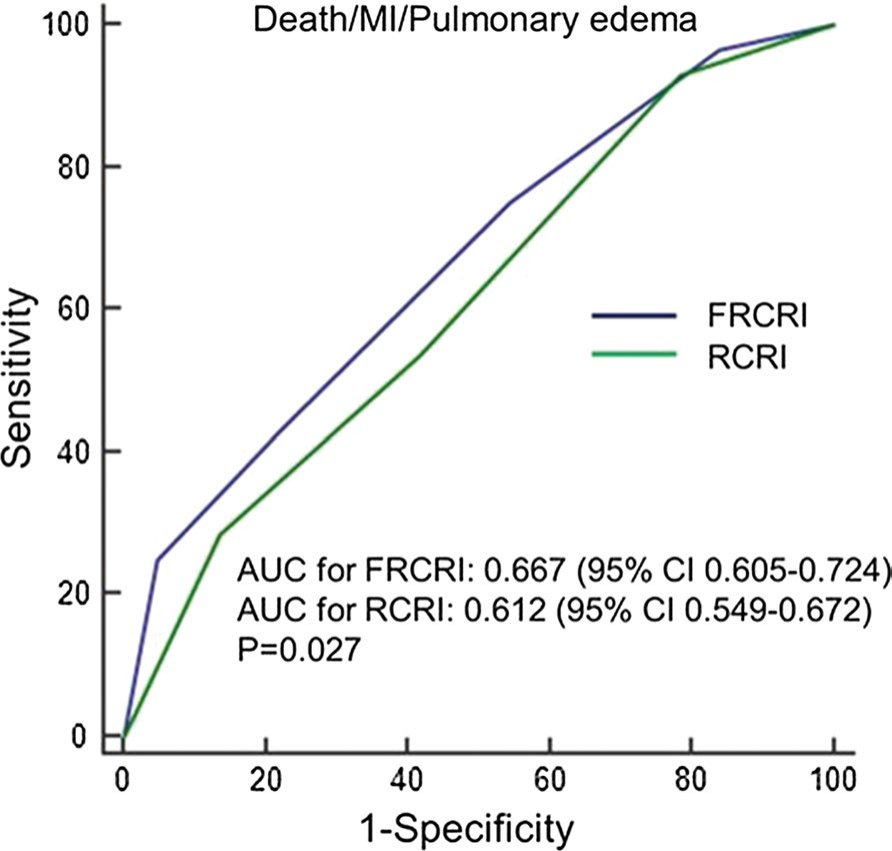
Receiver operating characteristic curve analysis of RCRI and FRCRI as predictors of perioperative MACE. RCRI = Revised Cardiac Risk Index, FRCRI = sum of Revised Cardiac Risk Index score and fragmented QRS score, MACE = major adverse cardiac events, MI = myocardial infarction, AUC = area under curve

**Fig. 5:**
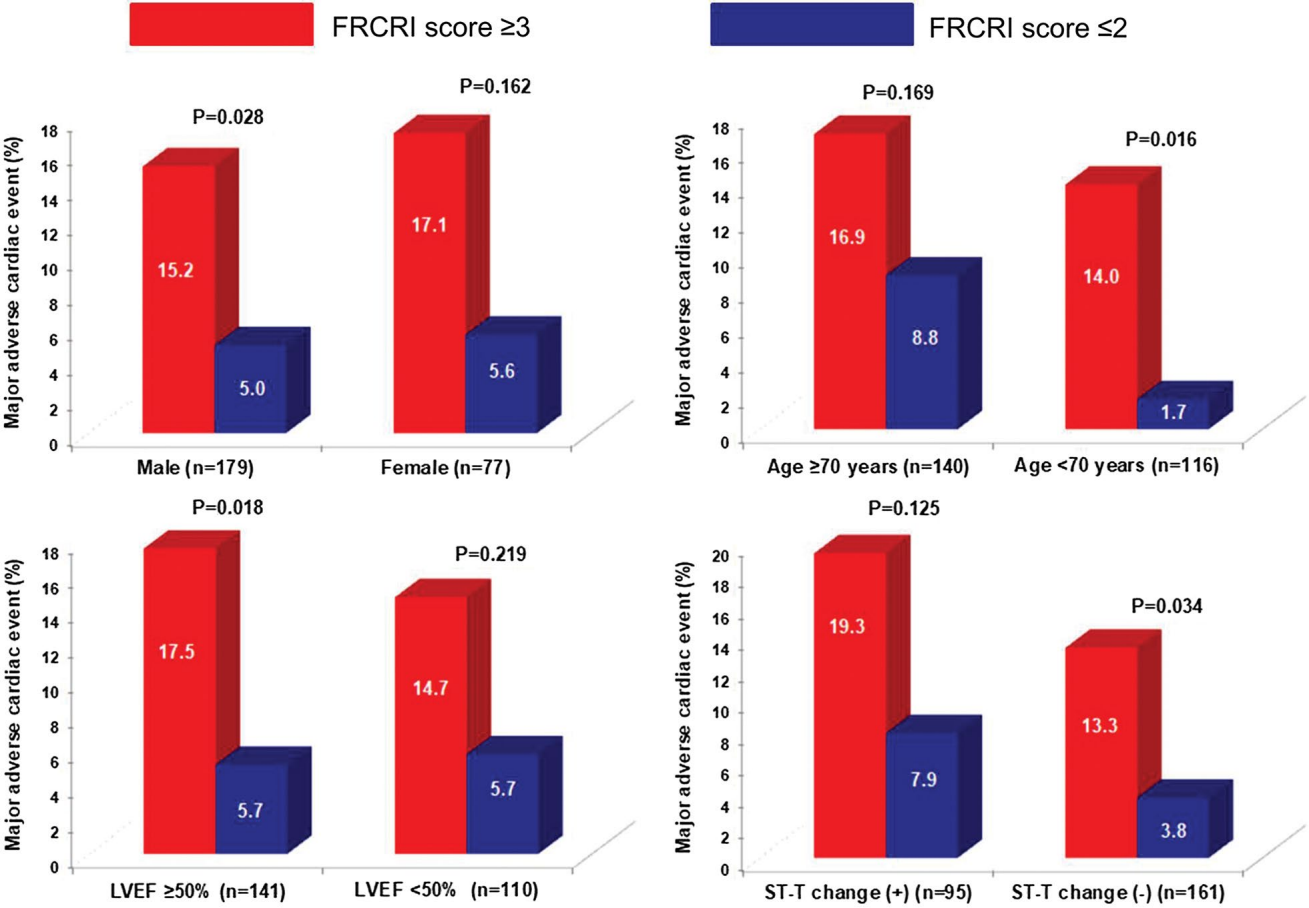
Subgroup analysis showing postoperative MACE according to the FRCRI score. *p* values for interaction according to sex, age, LVEF, and ST change were > 0.05. MACE = major adverse cardiac events, FRCRI = sum of Revised Cardiac Risk Index score and fragmented QRS score

The incidence of MACE according to RCRI score 1, 2, 3 and ≥ 4 were 3.9%, 11.3%, 11.3 and 18.9%, respectively. When the RCRI score was reclassified by summing the RCRI and the fQRS on ECG, the incidence rate of MACE according to FRCRI 1, 2, 3 and ≥ 4 were 0%, 7.3%, 14.6%, and 17.2%, respectively (Fig. 3), and 23 patients (32.4%) originally classified as RCRI 2 were reclassified as FRCRI 3. The area under the ROC curve for predicting postoperative MACE was 0.612 (95% CI 0.549 to 0.672) for RCRI. Adding fQRS to RCRI significantly increased the area under the curve to 0.667 (95% CI 0.605 to 0.724, *p* = 0.027) (Fig. 4). Subgroup analysis showed that FRCRI ≥ 3 was associated with postoperative MACE in men, and patients aged < 70 years, as well as LVEF ≥ 50%, or no ST-T segment changes on ECG (Fig. 5). However, statistical tests for interaction were not significant (*p* > 0.05).

## Discussion

In the present study, fQRS on a 12-lead ECG was correlated with myocardial perfusion defect in patients with severe renal dysfunction and was shown to be a valuable predictor of postoperative cardiac events after adjusting for the RCRI and age per year in patients with severe renal dysfunction undergoing noncardiac surgery. In addition, FRCRI score reclassified by summing the RCRI and the fQRS on ECG was found to be more powerful than RCRI score for postoperative cardiac risk stratification, and FRCRI ≥ 3 was predictor of postoperative MACE regardless of sex, age, LVEF, and ST-T changes on ECG.

Chronic kidney impairment is a known independent risk factor for postoperative death and cardiovascular events after elective noncardiac surgery [8]. In the previous meta-analysis, after abdominal aortic aneurysm repair, the pooled incidence of postoperative mortality was 3.2% in patients with normal renal function and 9.2% in patients with chronic kidney disease (pooled absolute risk increase of 4.7%) [8, 12–18]. Moreover, a graded relationship existed between the severity of preoperative renal impairment and the incidence of postoperative adverse events [8] and patients on dialysis were at the highest risk of such events [8]. Therefore, more reliable predictors of perioperative cardiac complications are required for patients with severe renal dysfunction who are vulnerable to postoperative cardiac complications. However, there have been few studies on predictive factors of postoperative cardiac complications in patients with severe renal dysfunction.

RCRI is a scoring system for predicting postoperative cardiovascular complications, and renal failure is one of the six risk factors that constitute the score system [4]. However, when the remaining risk factor is calculated among patients with severe renal dysfunction, the association between the RCRI score and the actual rate of MACE is likely to be compromised due to a decrease in available risk factors. In fact, in this study, the incidence of MACE was same in the RCRI 2 group, which was considered as intermediate risk and the RCRI 3 group, which was considered as high risk.

In the preoperative risk stratification of patients who were undergoing intermediate-risk noncardiac surgeries, coronary CT angiography evaluations showed additive value to RCRI [19]. However, some of the patients with renal failure has limited to undergo preoperative examinations that require the use of contrast agents such as coronary CT angiography due to the risk of renal toxicity. Therefore, a more simple and safe examination is needed for the evaluation of perioperative cardiac events in patients with severe renal dysfunction.

ECG is a very simple and safe diagnostic modality. Among the various ECG parameters, several studies have reported that fQRS is a sign of myocardial ischemia or scar [10, 11], and the number of leads with fQRS can help predict the prognosis of patients with acute myocardial infarction [20, 21]. In addition, previous study proved that perioperative MACE can be predicted when analyzed in addition to RCRI in patients undergoing vascular surgery [9]. In this study, FRCRI was also superior in predicting postoperative MACE compared to conventional RCRI. In particular, many of the patients with RCRI score 2 were reclassified to FRCRI score 3, and postoperative MACE was more common in FRCRI 3 than in FRCRI 2 group. From these results, the FRCRI score system in patients with severe renal dysfunction is believed to be helpful.

## Conclusions

The fQRS, which is simple and easily available through a 12-lead ECG, is an independent predictor of postoperative cardiac events in patients with severe renal dysfunction. Moreover, the newly reclassified FRCRI that incorporates fQRS, is a valuable predictor of postoperative MACE in these patients.

## Data Availability

The data used and/or analysed during the current study available from the corresponding author on reasonable request.

## Abbreviations

AUC: Area under curve
CI: Confidence interval
CHF: Congestive heart failure
ECG: Electrocardiogram
fQRS: Fragmented QR
FRCRI: Newly reclassified RCRI that incorporates fQRS
IHD: Ischemic heart disease
LVEF: Left ventricular ejection fraction
MACE: Major advanced cardiac events
OR: Odds ratio
RCRI: Revised Cardiac Risk Index
ROC: Receiver operating characteristic
SD: Standard deviation
SPECT: Single-photon emission computed tomography
